# CYP2C19^⁎^2 Polymorphism in Chilean Patients with In-Stent Restenosis Development and Controls

**DOI:** 10.1155/2017/5783719

**Published:** 2017-07-13

**Authors:** Jenny Ruedlinger, Yalena Prado, Tomás Zambrano, Nicolás Saavedra, Braulio Bobadilla, Marcelo Potthoff, Luis Pérez, Fernando Lanas, Luis A. Salazar

**Affiliations:** ^1^Center of Molecular Biology and Pharmacogenetics, Scientific and Technological Bioresource Nucleus, Universidad de La Frontera, Temuco, Chile; ^2^Department of Internal Medicine, Faculty of Medicine, Universidad de La Frontera, Temuco, Chile; ^3^Facultad de Medicina, Universidad de Concepción, Concepción, Chile

## Abstract

Clopidogrel is an antiplatelet drug especially used in patients undergoing percutaneous coronary interventions (PCI). Polymorphisms within* CYP2C19* can result in important interindividual variations regarding therapeutic efficacy. Therefore, we aimed to evaluate the impact of the CYP2C19^⁎^2 variant (rs4244285) on in-stent restenosis occurrence in Chilean patients who underwent PCI and received clopidogrel. A total of 77 cases with stenosis >50% in the angioplasty site (62.75 ± 9.8 years, 80.5% males) and 86 controls (65.45 ± 9.8 years, 72.1% males) were studied. The polymorphism was genotyped using TaqMan® Drug Metabolism Genotyping Assays. Overall, CYP2C19^⁎^2 allele frequency was 8.3%. Diabetes, chronic lesions, and bare metal stents (BMS) were observed more often in cases than in controls (*p* = 0.05,* p* = 0.04, and* p* = 0.02, resp.). Genotypic frequencies did not differ significantly between the groups (*p* = 0.15). Nonetheless, the mutated allele was observed in a greater proportion in patients without in-stent restenosis (*p* = 0.055). There was no significant association between the rs4244285 variant and the occurrence of in-stent restenosis after PCI (OR = 0.44; 95% CI: 0.19 to 1.04;* p* = 0.06). In summary, no association was identified between the CYP2C19^⁎^2 variant and the development of coronary in-stent restenosis.

## 1. Introduction

Clopidogrel is an antiplatelet drug widely used to reduce the risk of acute myocardial infarction and stroke, as well as for secondary prevention of atherosclerotic events in patients at high risk [1, 2]. This drug is often administered with aspirin as dual antiplatelet therapy (DAPT) in patients undergoing coronary angioplasty with stent implantation [3]. Clopidogrel is an inactive prodrug that requires liver activation by the cytochrome P450 enzyme complex, with CYP2C19 being one of the main enzymes involved in this process [4]. The active metabolite binds irreversibly to the P2Y12 receptor, inhibiting adenosine diphosphate- (ADP-) mediated platelet activation and aggregation [5].

*CYP2C19* polymorphisms have been previously associated with large interindividual variations in terms of therapeutic efficacy in patients treated with clopidogrel [6–8]. Different allelic variants of this gene have been identified, demonstrating that its activity may be normal, reduced, or increased according to the allele detected [9]. CYP2C19^*∗*^2 corresponds to a change of guanine by adenine at position 681 in exon 5 and results in a loss-of-function allele associated with decreased conversion of clopidogrel to its active metabolite, increased platelet reactivity during therapy, and poor clinical outcome, with a potential increase of in-stent thrombosis [6, 10]. On the contrary, the CYP2C19^*∗*^17 variant is associated with increased antiplatelet therapeutic response and a better clinical outcome [11–13]. Additionally, reports show that clopidogrel can prevent restenosis occurrence, partly due to the fact that inhibition of platelet activity would in turn decrease the release of platelet-derived growth factor (PDGF) [14, 15], which is a potent stimulator of vascular smooth muscle cell (VSMC) proliferation and cell migration [16].

Currently in the USA, patients with a poor metabolizer phenotype (^*∗*^2/^*∗*^2, ^*∗*^2/^*∗*^3, and ^*∗*^3/^*∗*^3) are advised to use drugs such as prasugrel and ticagrelor [17]; however, in Chile, information regarding the prevalence of CYP2C19 polymorphisms is still lacking. Since CYP2C19 is one of the main enzymes involved in hepatic bioactivation of clopidogrel, the polymorphic allele frequency of the CYP2C19^*∗*^2 variant was determined in Chilean patients with coronary disease, evaluating its role in the occurrence of in-stent restenosis.

## 2. Materials and Methods

### 2.1. Study Population

This work corresponds to an incident unpaired case-control investigation. The presence of the CYP2C19^*∗*^2 variant (rs4244285, 681G>A) was determined in 163 unrelated Chilean patients with coronary heart disease (CHD) admitted to the respective cardiology service of Dr. Hernán Henríquez Aravena Hospital (Temuco) or Dr. Guillermo Grant Benavente Hospital (Concepción), both in Chile. Patients underwent a successful PTCA and a coronary angiography control after 6 months, and all received DAPT. Patients with stenosis >50% were defined as cases and those with stenosis <50% were defined as controls by two experienced cardiologists. In total, both groups consisted of 77 cases and 86 controls. A record of demographic and anthropometric data, occurrence of myocardial infarction, presence of diabetes, biochemical tests (lipid profile, blood glucose, glycosylated hemoglobin, and serum creatinine), and smoking was elaborated, in addition to angiographic variables of the affected vessel, lesion characteristics, and stent type. The study protocol was approved by 2 independent ethics committees: Scientific Ethics Committee of Universidad de La Frontera and the Scientific Ethics Committee of the Health Service in Concepción. All individuals signed informed consent.

### 2.2. Samples Collection, DNA Extraction, and CYP2C19^*∗*^2 Genotyping

Blood samples used for biochemical determination and DNA extraction were obtained by venipuncture following 10–12 hours of fasting. Genomic DNA was extracted from leukocytes by a method optimized by Salazar et al. [18]. DNA quantification and purity were assessed by UV spectrophotometry using a microplate reader (Infinite® 200PRO NanoQuant, Tecan). The rs4244285 SNP was genotyped by Real-Time Polymerase Chain Reaction (RT-PCR) assay using the C_25986767_70 TaqMan Drug Metabolism Genotyping Assay (Life Technologies, CA, USA). Data were analyzed with the StepOne software v2.3. Genotyping was performed using the allelic discrimination plot issued after PCR amplification. No template controls (NTC) were included to assess the presence of contaminants.

### 2.3. Statistical Analysis

Data are presented as percentages and means ± standard deviation (SD). The differences between the means of two continuous variables were compared using two-tailed Student's *t*-test for unpaired samples when normal distribution of data was observed, according to D'Agostino testing, or by the nonparametric Mann–Whitney *U* test. To determine differences between noncontinuous variables, genotypic distribution, allele frequencies, and Hardy-Weinberg equilibrium, chi-square test (*χ*^2^) was used. The association between restenosis and the CYP2C19^*∗*^2 variant was estimated by calculating the odds ratio (OR) with a confidence interval (CI) of 95%. Additionally, a stratified Mantel-Haenszel analysis was performed to identify potentially confounding variables. A *p* value less than 0.05 was considered statistically significant.

## 3. Results

### 3.1. Clinical and Demographical Variables

Clinical, anthropometric, and laboratory characteristics of the studied subjects are shown in Table 1. The average age of the control group was 65.45 ± 9.8 years, whereas in the cases it was 62.75 ± 9.8 years (*p* = 0.08). A majority of male patients were observed in both groups (80.5% cases and 72.1% controls, *p* = 0.2). No significant differences regarding demographic parameters, smoking, body mass index (BMI), and blood pressure were observed. On the other hand, diabetes was more frequent in cases than in controls (42% and 27%, resp.; *p* = 0.05) (Table 1).

### 3.2. Angiographic Characteristics

Table 2 shows angiographic parameters of the study group according to the development of in-stent restenosis. Length (21.5 ± 5.4 mm in cases versus 21.4 ± 5.4 mm in controls, *p* = 0.92) and diameter (3.0 ± 0.45 mm in cases versus 2.9 ± 0.3 mm in controls, *p* = 0.95) values were comparable between the two groups. On the other hand, a higher proportion of subjects with chronic lesions developed restenosis than patients with acute lesions (*p* = 0.042). Moreover, when comparing stent types (BMS versus DES), the presence of BMS in the case group was significantly higher than in controls (87.0% versus 72.3%, *p* = 0.019).

### 3.3. CYP2C19^*∗*^2 and In-Stent Restenosis

Considering the total cohort, allele frequency for the CYP2C19^*∗*^2 variant was 8.3% (data not shown). Genotypic distribution and allelic frequencies for each group are shown in Table 3. Genotype frequencies observed were consistent with Hardy-Weinberg equilibrium in cases (GG: 89.6%; AG: 10.4%; AA: 0.0%, *χ*^2^ = 0.23, *p* = NS) and controls (GG: 79.0%; AG: 19.8%; AA: 1.2%, *χ*^2^ = 0.002, *p* = NS). The minor allele was present in a greater proportion in the control group versus cases (10% versus 5%, resp.). No significant differences were observed in genotype frequencies between patients with restenosis and controls (*p* = 0.15). A higher minor allele frequency (MAF) was observed in patients who did not develop in-stent restenosis, though not significant (*p* = 0.055), with an odds ratio of 0.44 (95% CI: 0.19 to 1.04; *p* = 0.06). Since no patients from the case group carried the homozygous variant, and just one individual presented this genotype in the control group, we were unable to study more profoundly the isolated effect of the AA genotype on restenosis development. However, we observed that individuals from the control group carrying the mutated allele and receiving clopidogrel developed significantly less in-stent restenosis during the study period, discarding diabetes as a possible confounding variable by a stratified and adjusted Mantel-Haenszel analysis.

## 4. Discussion

Sequence alterations in* CYP2C19* have been widely associated with therapeutic response variability to multiple drugs, as published by the Clinical Pharmacogenetics Implementation Consortium (CPIC) and reported as a warning by the FDA within Plavix (clopidogrel) indications [17]. It has been seriously recommended to consider* CYP2C19* genotypes in patients undergoing percutaneous coronary intervention (PCI), mainly due to the presence of loss-of-function alleles, which can result in subjects with a poor metabolizer phenotype who, therefore, should receive an alternative to clopidogrel therapy. This work constitutes the first study evaluating the association between the loss-of-function CYP2C19^*∗*^2 allele and the presence of in-stent restenosis in Chilean CAD patients. Our results show a MAF of 8.3% for the CYP2C19^*∗*^2 variant in the entire study group and 5% and 10% allelic distribution in cases and controls, respectively. Allelic frequency for this variant was previously reported to reach 12% for Chileans in a study including the general population [19]. Both frequencies are lower than observed for Caucasians and Africans (15%) or Asians (29–35%) [17]; however, our study reports a very similar frequency to countries such as Bolivia (7.8%) [20], Colombia (8.7%) [21], and Mexico (9.7%) [22] as well as Canadian native inhabitants (8.8%) [23]. Moreover, CYP2C19^*∗*^2 MAF obtained is comparable to different African populations such as Egyptians (10.9%) [24], Tanzanians (10%) [25], and Tunisians (10.5%) [26]; European populations such as Belgians (9.1%) [27]; and also Middle Eastern populations like Iranians (11.1%) [28] and Palestinians (9%) [29].

On the other hand, our results indicate a lack of association between the loss-of-function variant and restenosis development in the studied subjects. The relationship between low clopidogrel response and restenosis has been previously discarded in a study reporting that low platelet response to clopidogrel had no significant impact on restenosis after DES implantation [30]. However, there are reports indicating that there is a relationship with thrombus formation, raised by Oikawa et al. under the term “thromborestenosis,” mainly observed in sirolimus-eluting stents [31]. Nishio et al. [32] reported a higher incidence of target lesion revascularization (TLR) in patients classified as poor metabolizers, including genotype 2^*∗*^/2^*∗*^, and in intermediate metabolizers compared to extensive metabolizers (1^*∗*^/1^*∗*^) (*p* = 0.008). Sawada et al. [33] described that even though TLR is likely to be higher in carriers versus noncarriers of the 2^*∗*^ allele, it did not reach statistical significance (*p* = 0.06), which did occur when comparing the presence of subclinical thrombi, more frequent in the first group (*p* = 0.0002). Our data indicate a higher proportion of the mutated allele in patients who did not develop restenosis (*p* = 0.055; OR: 0.44, *p* = 0.06). Even though these results are similar to those reported by Nozari et al. [34], as they could not establish a higher risk between carrying the CYP2C19^*∗*^2 allele and in-stent restenosis occurrence, the elevated frequency of the mutated allele in controls rather than cases represents a controversial and unexpected finding, mainly due to the fact that several studies identify this polymorphism as a risk variant. However, various interethnic studies have also demonstrated the highly polymorphic nature of* CYP2C19*, which could partly explain the differences observed between different populations, especially for the higher MAF found in control subjects from our study. In line with this, a previous 2012 case-control investigation performed in the Korean population described that patients with essential hypertension (EH) had a lower frequency of the CYP2C19^*∗*^3 variant allele than did normal controls [35], which is similar to the particular allele distribution reported by our group. Thus, special attention must be paid to race, as it seems to play a determinant role for the interpretation of the studies involving this variant.

Contrasting our results, a Chinese investigation established that loss-of-function* CYP2C19* genotypes (^*∗*^2/^*∗*^2, ^*∗*^2/^*∗*^3, and ^*∗*^3/^*∗*^3) represent risk factors for restenosis in patients with stenting in the vertebral artery (*p* = 0.001) [36]. Another important factor to consider corresponds to the effect of clinical and angiographic variables. A significant difference was observed in the number of cases with diabetes mellitus versus controls. Although it has been well established that diabetes constitutes one of the main clinical predictors of restenosis [37–39], it has also been shown that diabetic patients have a lower response to clopidogrel and have lower levels of the circulating active metabolite [40], demonstrating also an increase in platelet reactivity and increased risk of thrombotic events [41]. In a recent study performed on patients with a coronary disease, it was determined that diabetics, CYP2C19^*∗*^2 phenotype carriers or both, required a 2–4-fold higher clopidogrel dose to obtain similar platelet reactivity to patients medicated with 75 mg having neither diabetes nor the loss-of-function variant [42]. With regard to the angiographic characteristics of the patients, a higher proportion of DES was observed in the control group, which has been abundantly associated with lower restenosis rates, namely, 5–10%, versus 20–30% when using BMS [43–45].

Among the limitations of our work, we can find the restricted sample size, creating the need to replicate this study in a larger cohort. Additionally, we cannot exclude that different* CYP2C19 *variants not included in this study are present, such as CYP2C19^*∗*^17, which in combination with a loss-of-function variant results in an intermediate phenotype and not in poor drug metabolism.

## 5. Conclusion

In summary, no association was found between the loss-of-function CYP2C19^*∗*^2 variant and in-stent restenosis development. Although our results provide valuable information regarding the prevalence of this variant in the Chilean population, the greater proportion of mutated alleles in controls versus cases is to some extent contradictory. However, this outcome should be interpreted with caution, since additional clinical and angiographic factors found in the case group may predispose importantly to restenosis development, particularly in the case of diabetics.

## Figures and Tables

**Table 1:** Clinical and demographic characteristics of the study group.

Parameter	Patients with restenosis	Controls	*p* value
	*n* = 77	*n* = 86	
Age (years)	62.75 ± 9.8	65.45 ± 9.8	0.08
Male (%)	80.5	72.1	0.20
BMI (kg/m^2^)	27.6 ± 3.8	28.0 ± 3.35	0.39
SBP (mmHg)	131.4 ± 22.2	135.0 ± 26	0.36
DBP (mmHg)	72.58 ± 14.0	72.47 ± 14.3	0.97
Heart rate (bpm)	69.4 ± 14.5	69.1 ± 14.3	0.37
Glucose (mg/dL)	135.3 ± 63.6	117.0 ± 42.5	0.14
Diabetes mellitus (%)	42	27	0.05
Dyslipidemia (%)	81	75	0.38
Total cholesterol (mg/dL)	159.0 ± 49.0	181.9 ± 128.0	0.26
Triglycerides (mg/dL)	160.4 ± 145.4	159.9 ± 85.8	0.12
LDL-C (mg/dL)	96.38 ± 54.0	99.40 ± 38.5	0.34
HDL-C (mg/dL)	40.78 ± 16.1	36.62 ± 7.9	0.48
Smoking (%)	77.8	69.0	0.29
Myocardial infarction (%)	45.6	32.9	0.12
Creatinine (mg/dL)	0.98 ± 0.28	1.02 ± 0.68	0.52
HbA1c (%)	6.58 ± 1.9	6.40 ± 1.25	0.23

Values expressed as mean ± standard deviation. SBP: systolic blood pressure; DBP: diastolic blood pressure; HDL-C: high-density lipoprotein-cholesterol; LDL-C: low-density lipoprotein-cholesterol; TG: triglycerides; HbA1c: glycosylated hemoglobin; HR: heart rate.

**Table 2:** Angiographic characteristics of the study group according to the development of in-stent restenosis.

Variable	Patients with restenosis	Controls	*p* value
	*n* = 77	*n* = 86	
*Lesion*			
Acute (%)	38.8	55.6	0.04
Chronic (%)	61.2	44.4	
Diameter (mm)	3.0 ± 0.45	2.9 ± 0.3	0.95
Length (mm)	21.5 ± 5.4	21.4 ± 5.4	0.92
*Lesion vessel (%)*			
LMCA	1.3	1	—
LAD	58.7	49	—
CX	13.3	20.4	—
RCA	26.7	29.6	—
*Stent type (%)*			
DES	13	27.7	0.02
BMS	87	72.3	

LMCA: left main coronary artery; LAD: left anterior descending artery; CX: circumflex artery; RCA: right coronary artery; BMS: bare-metal stent; DES: drug-eluting stent.

**Table 3:** Genotypic distribution, relative allele frequencies, and odds ratio (OR) for the CYP2C19^*∗*^2 variant in Chilean patients with and without restenosis.

Polymorphism rs4244285	Genotypes			Allele frequency	
	GG	AG	AA	G	A
Cases, *n* = 77	69 (89.6%)	8 (10.4%)	0 (0.0%)	0.95 (146)	0.05 (8)
Controls, *n* = 86	68 (79.0%)	17 (19.8%)	1 (1.2%)	0.90 (153)	0.10 (19)
*p* value	0.150 (*χ*^2^ = 3.76)			0.055 (*χ*^2^ = 3.66)	
Odds ratio	0.44 (95% CI: 0.19–1.04); *p* = 0.06				

*n*: number of individuals. Genotype frequency values given as percentage. *p* value calculated by chi-square. CI: confidence interval. HWE (cases: *χ*^2^ = 0.23; *p* = 0.63; controls: *χ*^2^ = 0.002; *p* = 0.95).
